# Probiotics treatment ameliorated mycophenolic acid-induced colitis by enhancing intestinal barrier function and improving intestinal microbiota dysbiosis in mice

**DOI:** 10.3389/fmicb.2023.1153188

**Published:** 2023-07-18

**Authors:** Pengpeng Zhang, Jinwen Chen, Yingzi Ming, Ying Niu

**Affiliations:** ^1^Organ Transplantation Center, The Third Xiangya Hospital of Central South University, Changsha, Hunan, China; ^2^Engineering and Technology Research Center for Transplantation Medicine of the National Ministry of Health, The Third Xiangya Hospital of Central South University, Changsha, Hunan, China

**Keywords:** mycophenolic acid, colitis, probiotic, intestinal microbiota, intestinal barrier

## Abstract

**Background:**

Mycophenolic acid (MPA)-induced colitis was still a severe side effect and challenge faced by solid transplant recipients. We aimed to explore the function and mechanism of probiotics in the treatment of MPA-induced colitis.

**Methods:**

In this study, 15 mice (C57BL/6) were randomly assigned into three groups: control (CNTL) group (*n* = 5), MPA group (*n* = 5) and the MPA + Probiotic group (*n* = 5). Bifid Triple Viable capsules, which were drugs for clinical use and consisted of *Bifidobacterium longum*, *Lactobacillus acidophilus*, and *Enterococcus faecalis*, were used in Probiotic group. Body weight change, stool scores, colon histopathology and morphology were used to evaluate the disease severity. The intestinal mucosal barrier function was assessed by measuring the expression level of secretory immunoglobulin A (sIgA), Zonula occludens-1 (ZO-1) and Occludin. Finally, 16S rDNA sequencing and bioinformatics analysis were performed on mice feces to compare the different intestinal microbial composition and diversity among three groups.

**Results:**

Compared with the CNTL group, the mice in MPA group showed a significantly decreased body weight, increased stool scores, shortened colon length and severe colon inflammation. However, probiotics treated MPA mice reversed the disease severity, indicating that probiotics ameliorated MPA-induced colitis in mice. Mechanistically, probiotics improved the intestinal barrier function by up-regulating the expression of sIgA, ZO-1 and Occludin. Moreover, MPA-induced colitis led to intestinal microbiota dysbiosis, including the change of *Firmicutes*/*Bacteroidetes* ratio, α- and β-diversity. But probiotic treated group showed mild change in these microbial features. Additionally, we found that *Clostridiales* was the most significantly different microbiota flora in MPA group.

**Conclusion:**

Probiotics treatment ameliorated MPA-induced colitis by enhancing intestinal barrier function and improving intestinal microbiota dysbiosis. *Clostridiales* might be the dominant functional intestinal microflora and serve as the potential therapy target in MPA-induced colitis.

## 1. Introduction

Mycophenolic acid (MPA) is a well-known immunosuppressive agent commonly used for prophylaxis of graft rejection following solid organ transplants, such as kidney, heart or liver transplant (Aliabadi et al., 2012; Di Maira et al., 2020; Jiang and Hu, 2021). However, MPA has been reported to have various gastrointestinal side effects, including diarrhea and colitis. And the morbidity of watery afebrile diarrhea caused by MPA is present in 36% of renal transplant recipients (de Andrade et al., 2014). Despite MPA-induced colitis showed relatively less occurrence in renal transplant recipients, there are still no well-established guidelines for management or treatment (Bani Fawwaz et al., 2021). Meanwhile, constant histologic features and diagnostic patterns of MPA-related colitis have not yet been established clearly (Liapis et al., 2013). From the clinicopathologic features of colitis, impaired intestinal mucosal barrier function and microflora have been shown to be often associated with colitis, accompanied by reduced expression levels of secretory immunoglobulin A (sIgA) and tight junction proteins (ZO-1 and Occludin; Yang et al., 2015; Li et al., 2018; Wang et al., 2019; Fu et al., 2021). Therefore, it is of great importance to develop effective methods to enhance intestinal mucosal barrier function and maintain intestinal microflora homeostasis for treatment of MPA-induced colitis.

Probiotics, including bacteria and yeast, are live microorganisms that have been largely demonstrated to manifest beneficial effects on human intestinal health. The application of probiotics as promise adjuvant treatment in various intestinal diseases have been also reported (Kim et al., 2019). A previous study revealed that probiotics have desirable effects in patients with inflammatory bowel disease (IBD), with improvement of both nutritional function and immune modulatory effects (Lorea Baroja et al., 2007). The effects of probiotics on human health also refer to the treatment of antibiotic-associated diarrhea and adjustment of immune system (Hempel et al., 2012; Bae et al., 2018). Moreover, Vibcenzo et al. reported that probiotics exhibited beneficial application in treatment of ulcerative colitis (Palumbo et al., 2016). Bifid Triple Viable capsules, which are consisted of *Bifidobacterium longum*, *Lactobacillus acidophilus*, and *Enterococcus faecalis*, are the most common probiotics for clinical use, especially in treatment of ulcerative colitis (Chen et al., 2019). Bifid Triple Viable may protect intestinal mucosa barrier, alleviate metabolic endotoxemia, thus improve chronic low-grade inflammation in liver and adipose tissue, and partially restore insulin sensitivity in high fat die mice by regulating gut microbiota (Lu et al., 2022). Moreover, Bifid Triple Viable ameliorate antibiotic-associated diarrhea by regulating the composition and structure of the gut microbiota in mice (Yang et al., 2022). But, in review of the literatures, the study between the probiotics, especially the Bifid triple viable, and MPA-induced colitis is limited.

Intestinal microbiota is considered as the largest symbiosis system in the body and numerous studies have shown its association with immune diseases, such as type II diabetes, IBD and multiple sclerosis (Lavelle and Sokol, 2020; Ghezzi et al., 2021; Yang et al., 2021). Several studies verified that Rhein and green tea polyphenol had the ability to modulate intestinal microbiota, which in turn ameliorated experimental-induced colitis (Wu et al., 2020, 2021). Meanwhile, a review indicated that probiotics could directly affect the intestinal microbiota by modulating its composition and functionality in obesity (Fontané et al., 2018). Therefore, the present study explored the relationship between probiotics, intestinal microbiota and MPA-induced colitis, providing a novel strategy for the treatment of MPA-induced colitis.

## 2. Materials and methods

### 2.1. Ethics approval

This research protocol was approved by the Committee on the Ethics of Animal.

Experiments of the Third Xiangya Hospital (no: 22207) and was conducted according to the Guidance for the Care and Use of Laboratory Animals of the National Institutes of Health.

### 2.2. Animal model and intervention

A total of 20 male SPF mice (9-week-old, C57BL/6) were provided by Hunan SLAC Laboratory Animals (Hunan, China). All mice were housed in a standard room with *ad libitum* water, rodent food and a 12/12 h light/dark cycle for 2 weeks.

After an acclimatization period, 15 mice were randomly divided into three groups: the Control (CNTL) group (*n* = 5), the MPA group (*n* = 5) and the MPA + Probiotic group (*n* = 5). In MPA group, 0.2 mL MPA solution (0.5 g/kg/day) was given to the mice by gavage at 8:00 am for 3 weeks, according to a previous study (Watanabe et al., 2006). In MPA + Probiotic group, 0.2 mL MPA solution (0.5 g/kg/day, at 8:00 am) and 0.2 mL Bifid Triple Viable suspension (25 mg/kg/day, at 4:00 pm) were given to the mice by gavage for 3 weeks. Bifid Triple Viable capsules are purchased from Shanghai Sine Pharmaceutical, which are drugs for clinical use and consisted of *Bifidobacterium longum* (≥1 × 10^7^ CFU/g), *Lactobacillus acidophilus* (≥1 × 10^7^ CFU/g), and *Enterococcus faecalis* (≥1 × 10^7^ CFU/g). In CNTL group, the same volume of 0.9% NaCl was given to the mice by gavage for 3 weeks. All mice were sacrificed and their colon were collected for further analysis.

### 2.3. Evaluation of disease severity

The mice were checked daily for MPA-induced colitis based on body weight and stool scores. Stool scores were used to assess the severity of diarrhea based on the previous study: 0, normal stools; 1, slight diarrhea with wet and soft stools; 2, moderate diarrhea with unformed stools and mild perianal stains; and 3, severe diarrhea with watery stools and severe perianal stains (Yang et al., 2022).

### 2.4. Colon histopathology and hematoxylin–eosin staining

Firstly, the length of colon was measured. Then a colon segment was fixed in 10% neutralized buffered formaldehyde at 4°C for 48 h and embedded in paraffin. The paraffin blocks were sliced into 4 μm sections. Paraffin sections were deparaffinized with xylenes and rehydrated by washing through a graded alcohol series to deionized water. The sections were stained by hematoxylin for 2 min and eosin for 30s and washed by warm water for 5 min. Then the sections were dehydrated by washing through a graded alcohol series to xylenes and mounted with cytoseal (8310, Thermo scientific, United States). The images were obtained using Leica Microsystems DMI 3000B observer. The Chiu’s scores were used to assess the pathological degree of colitis according to the previous study (Shin et al., 2014). These criteria were scored as follows: inflammation severity (0: none, 1: slight, 2: moderate, or 3: severe), extent of injury (0: none, 1: mucosal, 2: mucosal and submucosal, or 3: transmural), and crypt damage (0: none, 1: damage to the basal third of the crypt, 2: damage to the basal two-thirds of the crypt, 3: only surface epithelium intact, or 4: loss of entire crypt and epithelium).

### 2.5. Enzyme linked immunosorbent assay for sIgA

Total protein was extracted from the colon samples using a RIPA lysis buffer (AWB0139a, Abiowell, Changsha) with protease inhibitors cocktail (AWH0644a, Abiowell, Changsha). After detecting protein concentration by BCA assay kit, sIgA ELISA kit (CSB-E08413m, CUSABIO, Wuhan) was used to measure the concentration of sIgA in colons according to the instruction.

### 2.6. Western blot analysis for ZO-1 and Occludin

Total protein was extracted from the colon samples using a RIPA lysis buffer with protease inhibitors. The BCA assay kit was used to measure protein concentration. Equal amounts of proteins were loaded and separated by SDS-Poly-Acrylamide Gel Electrophoresis (PAGE). Proteins were transferred to 0.2 μm nitrocellulose membrane (1620112, Bio-Rad, United States). Membranes were incubated with Tris buffered saline containing 0.1% Tween-20 (TBST) and 5% skim milk for 1 h at room temperature, followed by an incubation with primary antibodies against ZO-1 (21773-1-AP, Proteintech, United States), Occludin (13409-1-AP, Proteintech, United States) and β-actin (66009-1-Ig, Proteintech, United States) for overnight at 4°C. After washing with TBST buffer, the membranes were incubated with secondary antibody for 2 h at room temperature on next day. Then the protein bands were visualized using an ECL kit by Bio-rad ChemiDoc XRS plus an image analyzer (Bio-Rad, Hercules, CA, United States) after TBST washing.

### 2.7. Quantitative real-time polymerase chain reaction

Total RNA was extracted from colon tissues using TRIzol reagent (Invitrogen, Carlsbad, CA). Extracted total RNA (1,000 ng) was used as a template for reverse transcription into cDNA using Reverse Transcript Reagents kit (Roche Molecular Systems, Branchburg, NJ). Synthesized cDNA was mixed with iTaq Universal SYBR green supermix (Bio-Rad Laboratories, Hercules, CA) and amplified by a real time ABI 7500 PCR system (Applied Biosystems, Foster City, CA) with the primers. The data were analyzed using the 2^−ΔΔCt^ method, where the GAPDH was used as an endogenous control gene. The primer sequences were listed in the Table 1.

**Table 1:** The primer sequences list of qPCR.

Gene name	Note	Sequence
ZO-1	Forward	5’-GCCGCTAAGAGCACAGCAA-3’
	Reverse	5’-GCCCTCCTTTTAACACATCAGA-3’
Occludin	Forward	5’-TGAAAGTCCACCTCCTTACAGA-3’
	Reverse	5’-CCGGATAAAAAGAGTACGCTGG-3’
GAPDH	Forward	5’-GGAGCCAAACGGGTCATCAT −3’
	Reverse	5’-CTCGTGGTTCACACCCATCA-3’

### 2.8. 16S rDNA sequencing

#### 2.8.1. rDNA extraction

Total genomic DNA from fecal samples was extracted using CTAB/SDS method. DNA concentration and purity were measured on 1% agarose gels. According to the concentration, DNA was diluted to 1 ng/μl using sterile water. Primer:16S V3-V4: 341F-806R, 18S V9: 1380F-1510R, ITS1: ITS1F- ITS2R. 16S /18S rRNA genes were amplified using the specific primer with the barcode. All PCR reactions were carried out in 30 μL reactions with 15 μL of Phusion® High-Fidelity PCR Master Mix (New England Biolabs), 0.2 μM of forward and reverse primers, and about 10 ng template DNA.

#### 2.8.2. PCR products quantification and qualification

We mixed the same volume of 1X loading buffer (contained SYB green) with PCR products and ran electrophoresis on 2% agarose gel for detection. Samples with bright main strip between 400 and 450 bp were chosen for further experiments.

#### 2.8.3. Library preparation and sequencing

Sequencing libraries were generated using TruSeq® DNA PCR-Free Sample Preparation Kit (Illumina) following manufacturer’s recommendations and index codes were added. The library quality was assessed on the Qubit@ 2.0 Fluorometer (Thermo Scientific) and Agilent Bioanalyzer 2,100 system. At last, the library was sequenced on an Illumina NovaSeq6000 platform and 250 bp paired-end reads were generated.

#### 2.8.4. Sequencing data analysis

Paired-end reads from the original DNA fragments were merged using FLASH (Magoč and Salzberg, 2011). Paired-end reads were assigned to each sample according to the unique barcodes. Sequences analysis were performed by UPARSE software package using the UPARSE-Operational Taxonomic Units (OTU) and UPARSE-OTUref algorithms (Edgar, 2010; Edgar, 2013). α and β diversities were estimated using MOTHUR software (v1.31.2) and the QIIME pipeline (v1.8.0), respectively (Schloss et al., 2009; Caporaso et al., 2010). There were six indexes for evaluating α-diversity, including Observed Species, Goods coverage, Chao1, ACE, Shannon and Simpson. Shannon curves were generated based on these indexes. β-diversity analysis was calculated by unweighted UniFrac (Lozupone et al., 2011) and visualized by Principal co-ordinates analysis (PCoA) downscaling. LefSe analysis was used to count species with significant differences in community structure between sample groups and the linear discriminant analysis (LDA) score > 2 was considered as significant difference, which was used to screen for characteristic biomarkers of the intestinal microbiota between groups (Segata et al., 2011; Langille et al., 2013).

### 2.9. Statistical analysis

All data were expressed as mean ± standard error of mean (SEM). The comparison between two groups was performed using unpaired Student’s *t*-test. The comparison between multiple groups was performed using one-way ANOVA. Receiver operation characteristic (ROC) analysis to predict the diagnostic efficiency of the different intestinal microbiota was done by SPSS (v 25.0; SPSS, IL, United States). The value of *p* < 0.05 was considered statistically significant.

## 3. Results

### 3.1. Probiotics ameliorate MPA-induced colitis in mice

**Figure 1:**
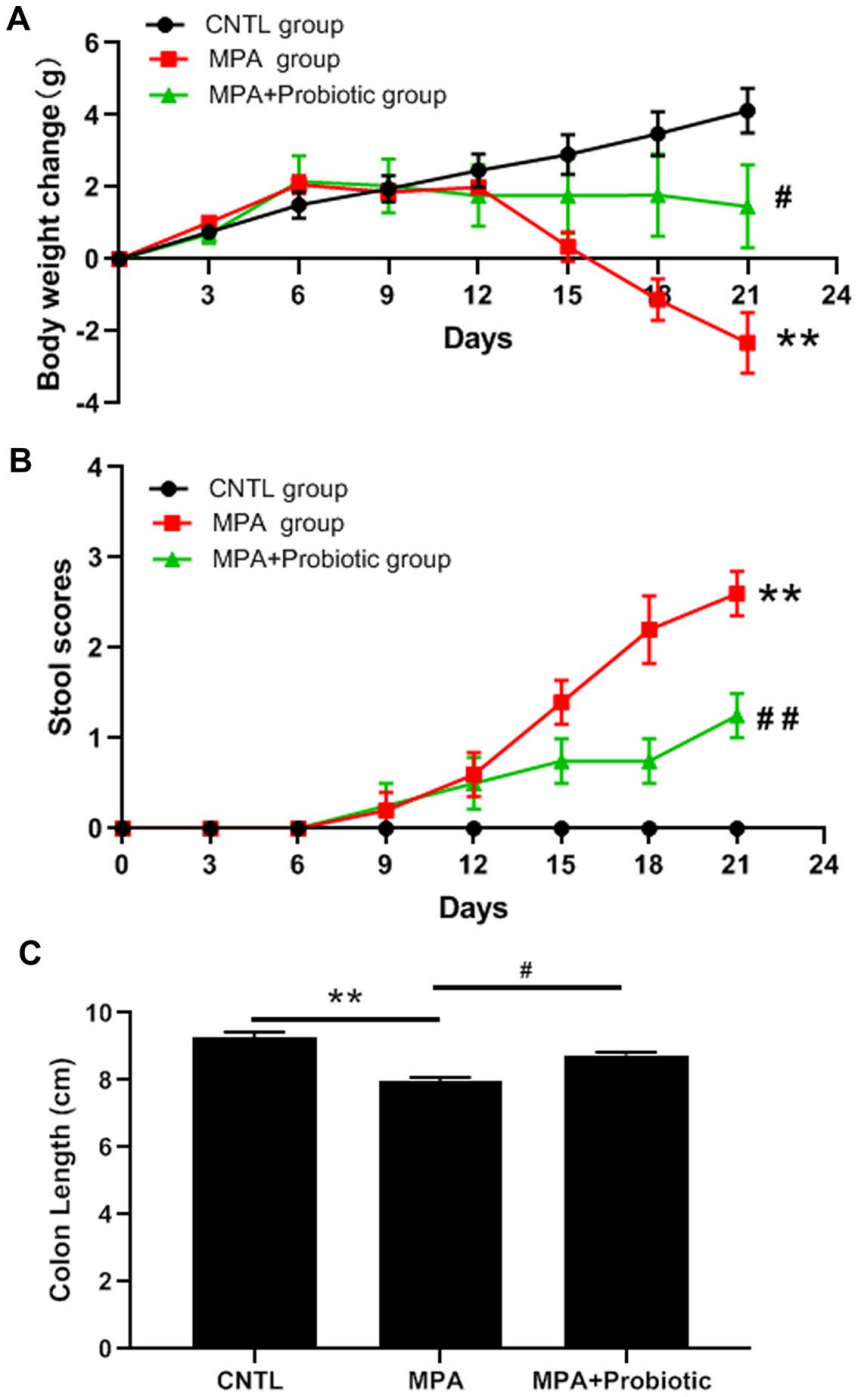
Effects of MPA and probiotic on body weight change **(A)**, stool scores **(B)** and colon length **(C)**. **p < 0.01 (CNTL group vs. MPA group); #*p* < 0.05 and ##*p* < 0.01 (MPA group vs. MPA + Probiotic group).

We first evaluated the disease severity of MPA-induced colitis based on body weight, stool scores, colon length and histopathology. According to the body weight results, there was no significant difference among three groups on the first 12 days. Subsequently, the mice in MPA group exhibited a significantly decreased body weight compared with CNTL group from the 15 days (*p* = 0.032) and probiotics treatment reversed mice phenotype (Figure 1A). From the stool scores results, the mice in each group showed the normal stool on the first 6 days. Subsequently, the mice in MPA group exhibited a significantly increased stool scores compared with CNTL group from the 15 days (*p* = 0.008), which was also reversed by probiotics treatment (Figure 1B).

**Figure 2:**
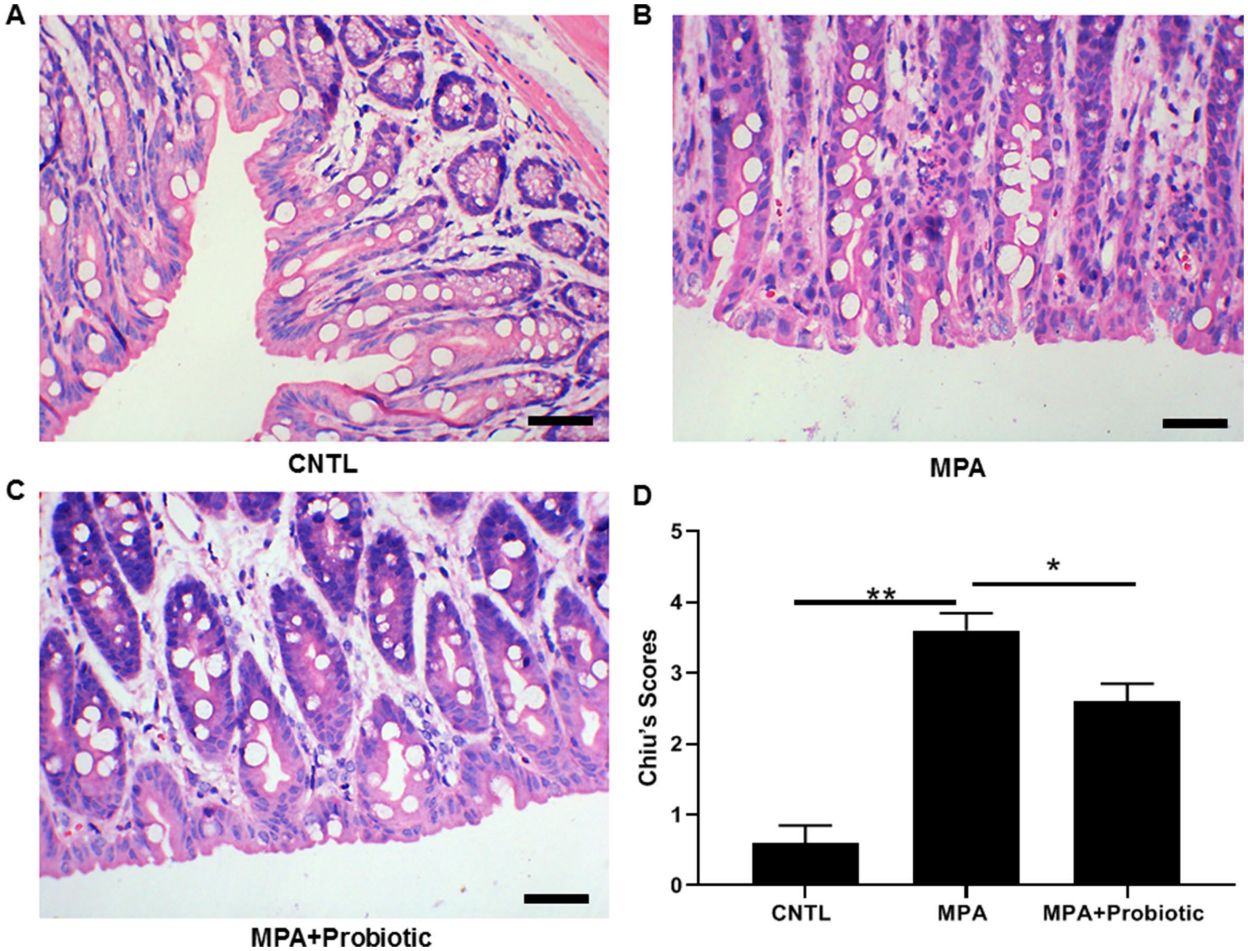
The H&E staining of colon tissue among three groups. **(A)** Normal colon tissue in CNTL group; **(B)** MPA induced severe colitis with structural damage and massive inflammatory cell infiltration; **(C)** Probiotic treatment improved the severity of MPA-induced colitis; **(D)** The statistical analysis for three groups (*p < 0.05, **p < 0.01, Scale bar = 20 μm).

As shown in Figure 1C, the colon length in MPA group was shorter than that in CNTL group (*p* = 0.006). But the colon length in probiotic treatment group became longer than that in MPA group (*p* = 0.015). In addition, the H&E staining showed that MPA induced severe colitis, accompanied by damaged colon structure and massive infiltration of inflammatory cells. While probiotic treatment improved the severity of MPA-induced colitis (Figure 2).

Taken together, our results suggested that probiotics significantly ameliorated MPA-induced colitis in mice.

### 3.2. Probiotics improve the intestinal barrier function by up-regulating the expression of sIgA, ZO-1 and Occludin

**Figure 3:**
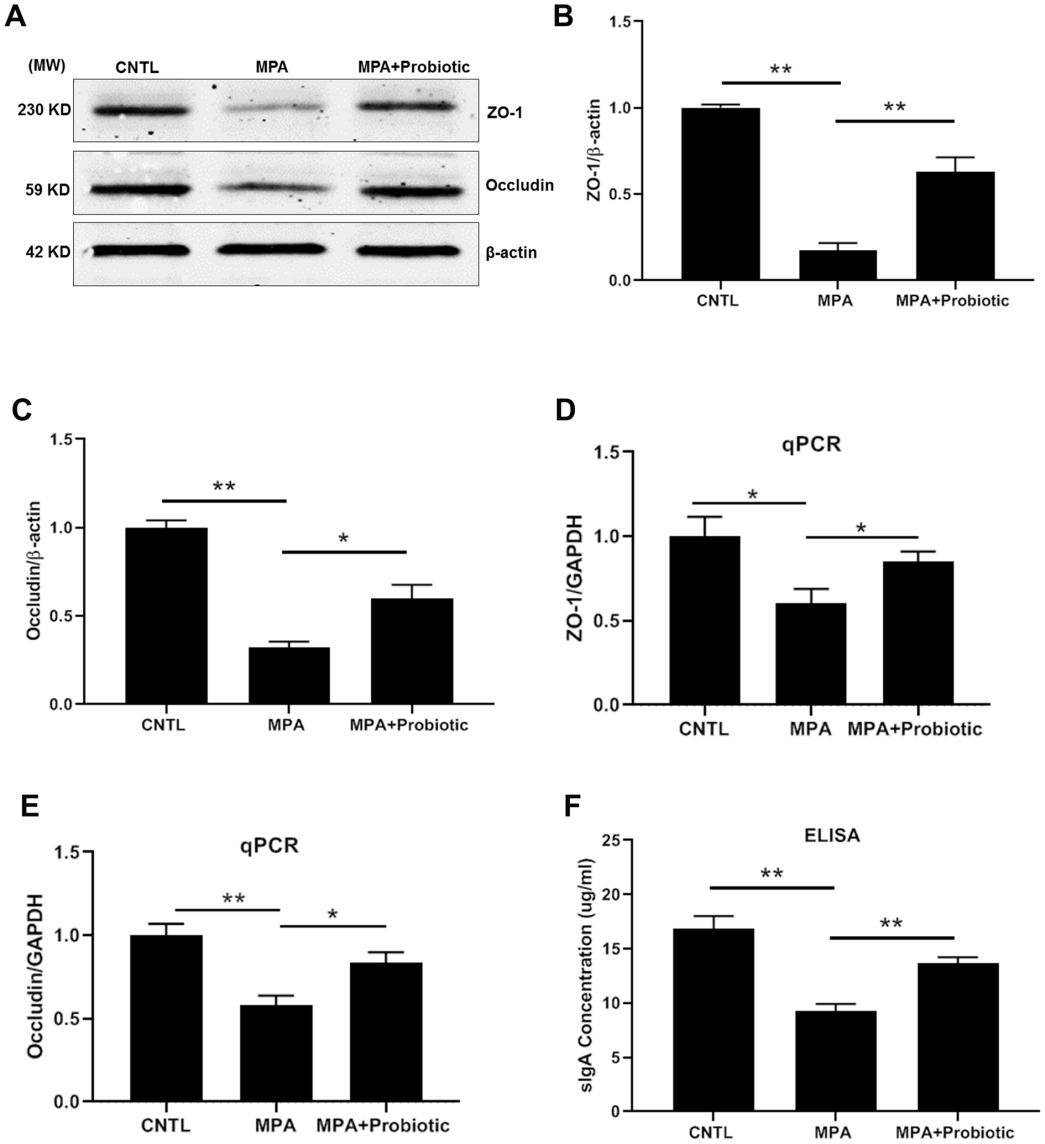
The expression level of ZO-1, Occludin and sIgA among three groups. **(A)** The representative image of Western blot of ZO-1 and Occludin; **(B,C)** The protein level of ZO-1 and Occludin among three groups by Western blot; **(D,E)** The mRNA expression level of ZO-1 and Occludin among three groups by qPCR; **(F)** The expression level of sIgA among three groups by ELISA (*p < 0.05, **p < 0.01).

We then examined the expression level of ZO-1, Occludin and sIgA to assess the intestinal barrier function. As shown in Figures 3A–E, both the protein and mRNA expression level of ZO-1 and Occludin were decreased in MPA group compared with the CNTL group. At the same time, the expression level of sIgA were also decreased in MPA group compared with the CNTL group by ELISA (Figure 3F). However, probiotic treatment up-regulated the expression level of ZO-1, Occludin and sIgA, suggesting the improvement of intestinal barrier function.

### 3.3. Probiotics improve the intestinal microbiota dysbiosis in MPA-induced colitis

**Figure 4:**
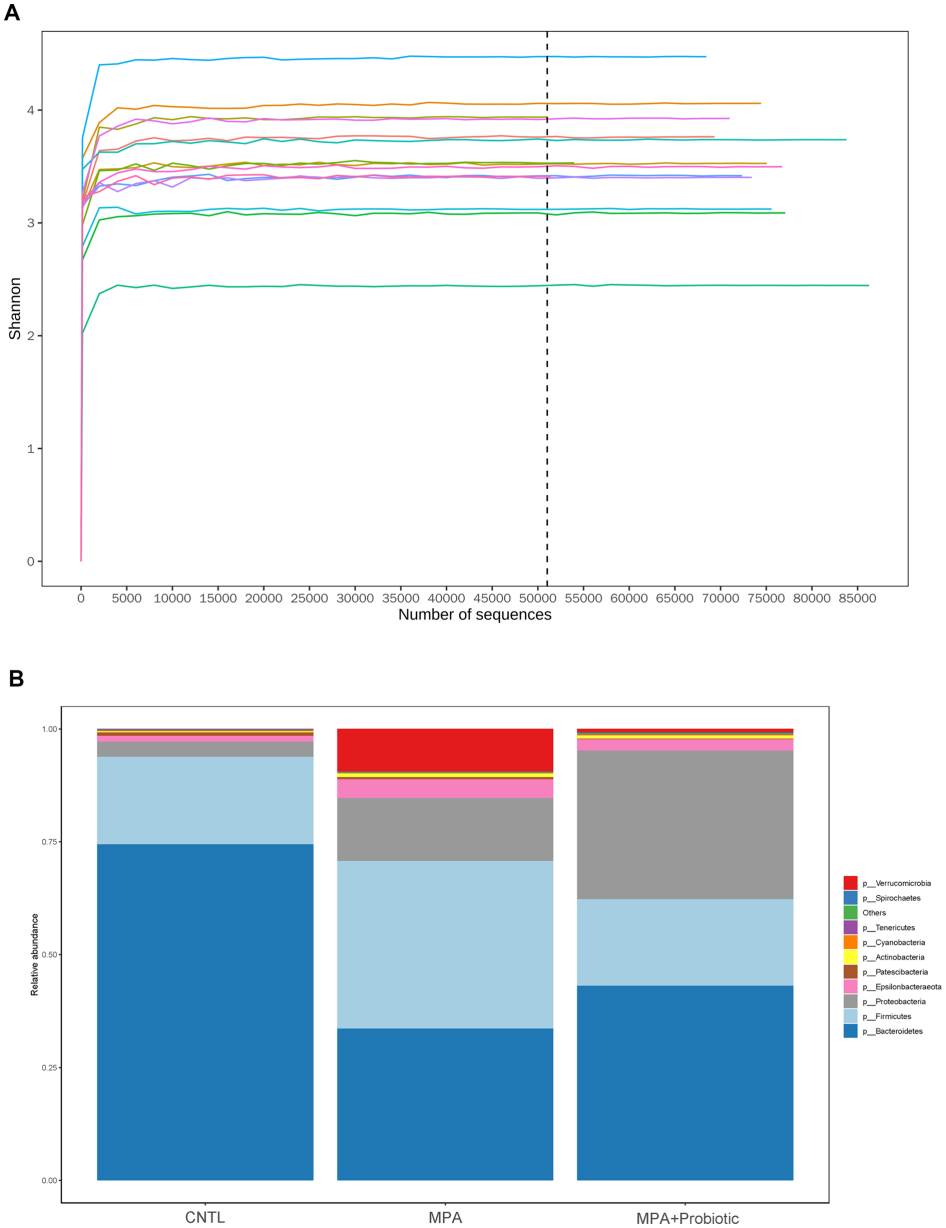
**(A)** The Shannon curve from each sample; **(B)** the relative abundance of intestinal microbiota among three group at the phylum levels.

The Shannon curve showed that our 16 s DNA sequencing data detected the most microbial information from each sample (Figure 4A). Thereafter, we assessed the relative abundance of intestinal microbiota among three groups at the phylum levels. As shown in Figure 4B and Table 2, and probiotic treatment reduced the change of microbial properties as that in MPA group. In detail, *Bacteroidetes* was the most common phyla in CNTL group, accounting for 74.4% of abundance. However, the relative abundance of *Bacteroidetes* decreased to 33.6% in MPA group. After probiotics treatment, the relative abundance of *Bacteroidetes* increased to 43.1%. Moreover, the relative abundance of *Firmicutes* in MPA group (37.2%) was significantly higher than that in CNTL group (19.4%). But the relative abundance of *Firmicutes* in probiotic group decreased to 19.2%. We also evaluated the *Firmicutes*/*Bacteroidetes* ratio among three groups, which was 0.26 in CNTL group, 1.11 in MPA group and 0.45 in Probiotic group.

**Table 2:** The relative abundance of top 10 Phylum groups.

Phylum name	CNTL	MPA	MPA + Probiotic
p__*Bacteroidetes*	0.744	0.336	0.431
p__*Firmicutes*	0.194	0.372	0.192
p__*Proteobacteria*	0.034	0.139	0.329
p__*Verrucomicrobia*	0.001	0.095	0.008
p__*Epsilonbacteraeota*	0.012	0.041	0.026
p__*Actinobacteria*	0.002	0.009	0.007
p__*Patescibacteria*	0.008	0.005	0.001
p__*Cyanobacteria*	0.001	0.001	0.002
p__*Spirochaetes*	0.001	0.000	0.002
p__*Tenericutes*	0.001	0.001	0.001
Others	0.001	0.002	0.002

**Figure 5:**
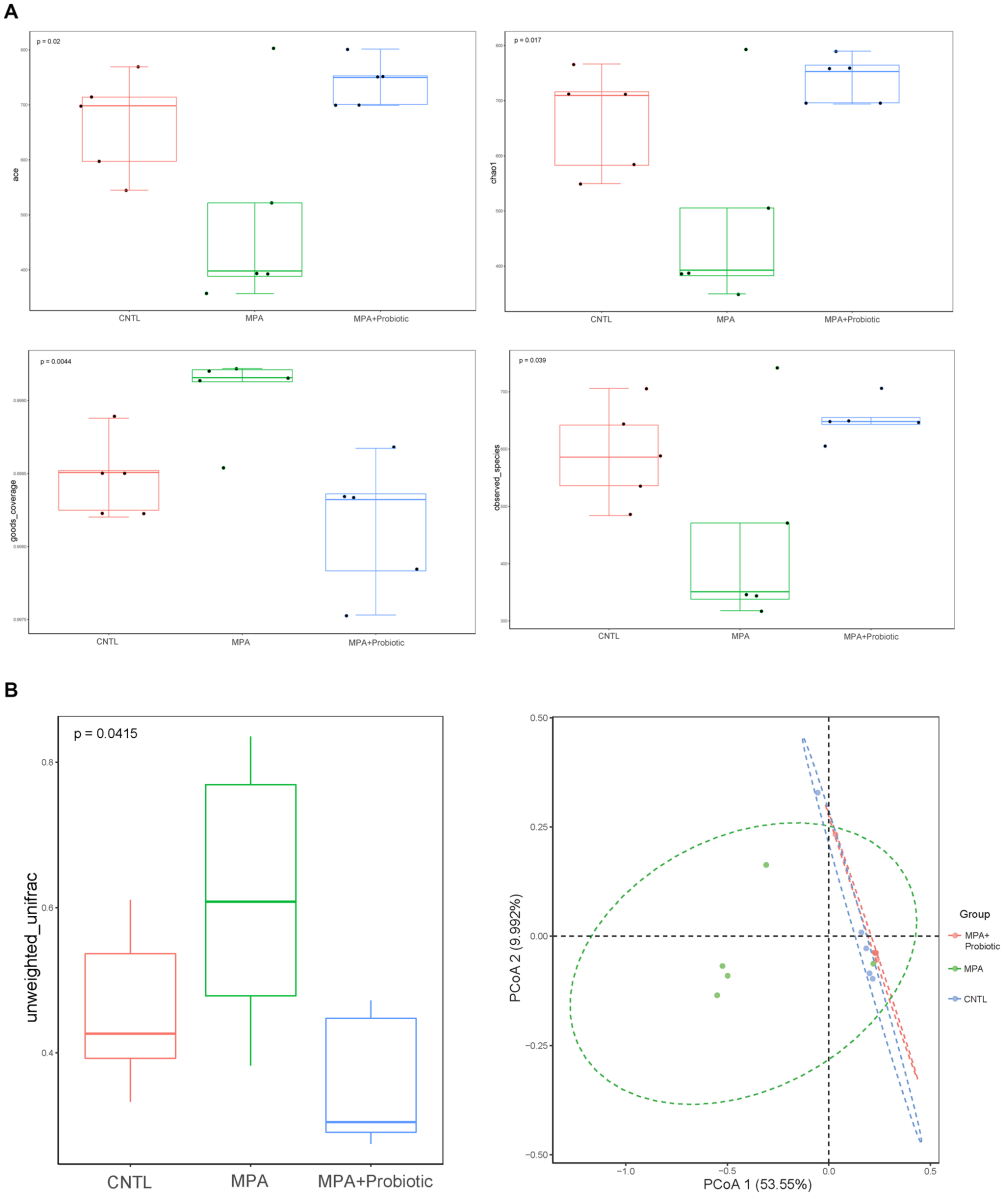
**(A)** The α-diversity (ace index, chao1 index, goods_coverage index and observed_species index) among three groups; **(B)** the β-diversity and PCoA analysis in unweighted UniFrac distances among three groups.

The α-diversity is the diversity of species within a given community, which represented the richness of species in each group. Six indexes were used for evaluating α-diversity in our data. Our results showed that there were differences on ace index (*p* = 0.02), chao1 index (*p* = 0.017), goods_coverage index (*p* = 0.004) and observed_species index (*p* = 0.039) among three groups (Figure 5A). We further analyzed β-diversity to assess the structure of the intestinal microbiota community. As shown in Figure 5B, there were significant differences in unweighted UniFrac distances analysis among three groups (*p* = 0.042) and probiotics treatment could reduce the difference in unweighted UniFrac distances between CNTL and MPA group. In addition, the PCoA analysis exhibited the same result among three groups (Figure 5B). These data indicated that probiotics treatment could reverse the change of intestinal microbiota in MPA-induced colitis and probiotics might ameliorate MPA-induced colitis by improving intestinal microbiota dysbiosis.

**Figure 6:**
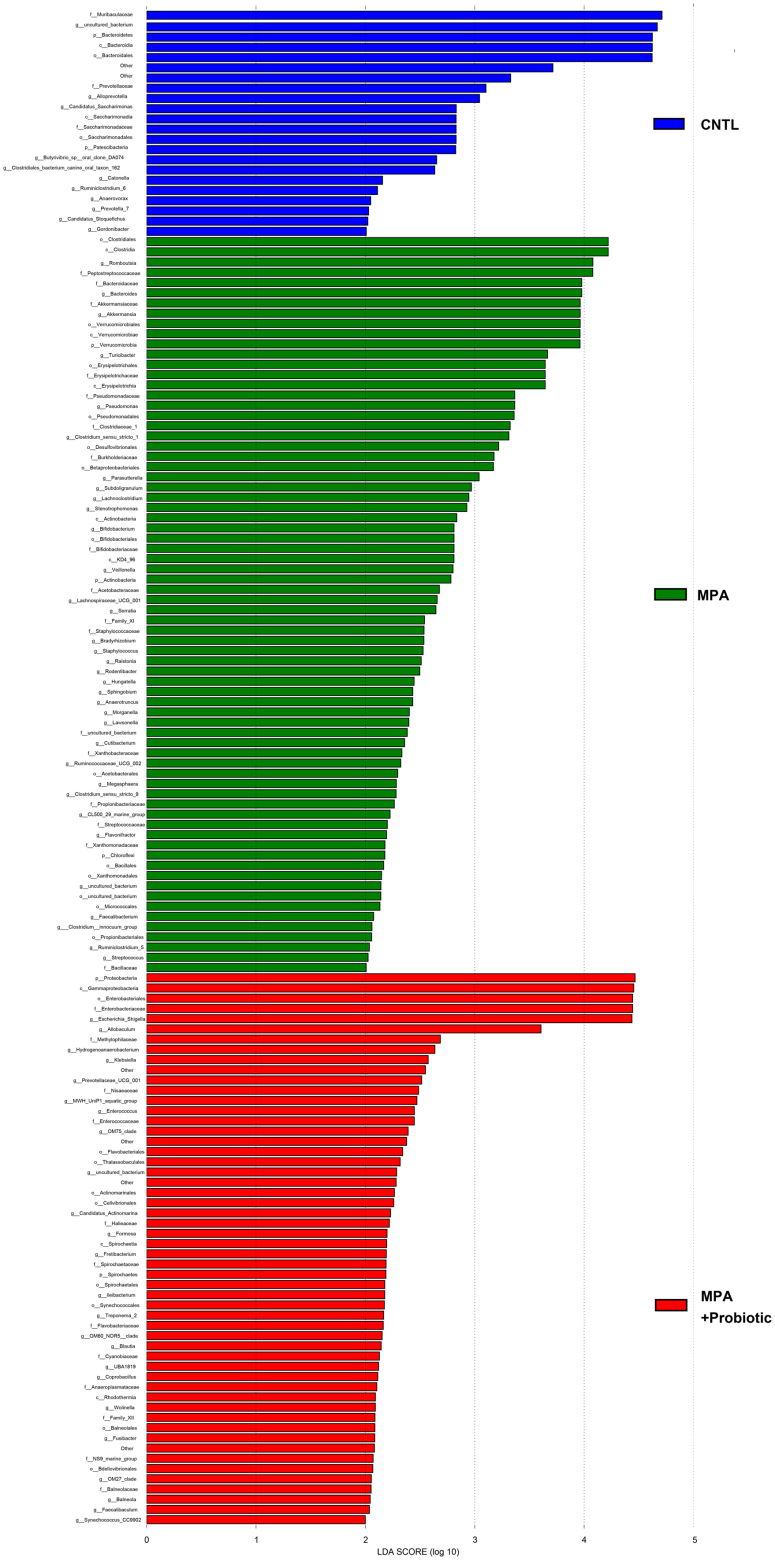
The LefSe analysis to explore the most significantly different microbiota flora among three groups.

**Figure 7:**
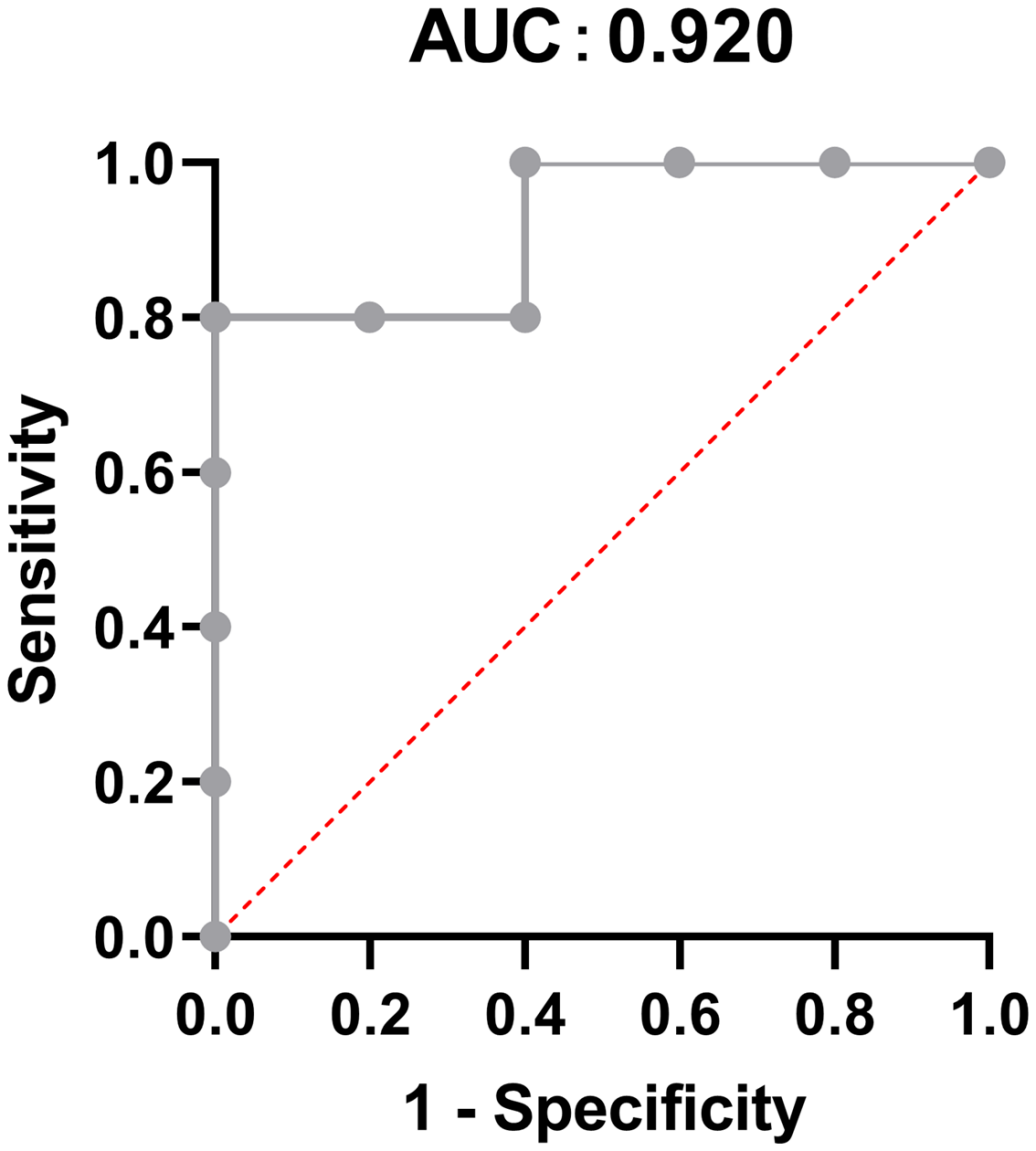
The Receiver operation characteristic (ROC) analysis to predict the diagnostic efficiency of the *Clostridiales* for MPA-induced colitis.

To explore the most significantly different microbiota flora in MPA group, we performed LefSe analysis. Compared with the CNTL group and Probiotic group, we found that *Clostridiales* flora, belonging to *Firmicutes* phylum, was significantly increased in MPA group (Figure 6). We also did ROC analysis to explore the diagnostic efficiency of the *Clostridiales* for MPA-induced colitis and the area under the curve (AUC) of the *Clostridiales* was 0.920 (Figure 7).

## 4. Discussion

MPA-induced colitis is still a severe side effect and challenge faced by solid transplant recipients, which, to date, was lack of effective treatments. Though probiotics exhibit desirable benefits in treatment of ulcerative colitis, no reports explore its function in MPA-induced colitis. In this present study, we found that probiotics have the ability to ameliorate MPA-induced colitis by enhancing intestinal mucosal barrier function and improving intestinal microflora dysbiosis.

According to the previous study reported by Watanabe, we successfully established the MPA-induced colitis model (Watanabe et al., 2006). Compared with CNTL group, the mice in MPA group suffered significant body weight loss, increased stool scores and severe colitis. However, probiotics treatment reversed these syndromes, indicating that probiotics could attenuate the MPA-induced colitis in mice.

Increasing evidences demonstrated that intestinal mucosal barrier dysfunction was critical in Ulcerative Colitis development and tight junction proteins (ZO-1 and Occludin), the key components of intestinal barrier, played important roles in the maintenance of intestinal permeability and integrity (Tan and Zheng, 2018). Our results showed that the expression level of ZO-1, Occludin and sIgA were significantly decreased in MPA group compared with the CNTL group. However, probiotic treatment up-regulated the expression of ZO-1, Occludin and sIgA to improve the intestinal barrier function. A previous study reported that dietary threonine supplementation enhanced intestinal barrier function by regulating the synthesis of intestinal tight junction proteins (Azzam et al., 2011), which was in consistent with our results. Another study also reported that dietary supplementation with *Clostridium butyricum* improved the intestinal barrier function of weaned piglets upon enterotoxigenic *Escherichia coli* K88 infection. Taken together, our results indicated that probiotics could ameliorate MPA-induced colitis, but the detailed mechanism awaited future investigation.

It has been known that probiotics have the ability to shape the intestinal microbiota composition, leading to control of multiple intestinal bowel diseases and further affecting the overall host health (Kim et al., 2019). Several studies confirmed that intestinal microbiota were capable of ameliorating experimental colitis (Wu et al., 2020, 2021). We found that probiotics ameliorate MPA-induced colitis by modulating intestinal microbiota. Our results showed that the species composition and relative abundance of the intestinal microbiota in MPA group changed significantly, including the percentage of *Bacteroidetes* and *Firmicutes* phylum, α- and β-diversity. While probiotic treatment was capable of reducing those changes as that in MPA group. *Bacteroidetes* and *Firmicutes* are two major phyla in normal human intestinal microbiota comprises (Jandhyala et al., 2015). A recent study indicated that *Bacteroidetes* species were correlated with disease activity in ulcerative colitis and the loss of key *Bacteroidetes* species might exacerbate ulcerative colitis (Nomura et al., 2021). Moreover, the *Firmicutes*/*Bacteroidetes* ratio is associated with colitis. For example, Baicalin has been used to treat ulcerative colitis by decreasing the *Firmicute/Bacteroidetes* ratio (Zhu et al., 2020). *Clostridium butyricum* also decreases *Firmicutes/Bacteroidetes* ratio to reduce colitis associated with colon cancer in mice (Liu et al., 2020). In our study, the *Firmicutes/Bacteroidetes* ratio in MPA group was 1.11, which was significantly higher than that in CNTL group (0.26). However, the *Firmicutes/Bacteroidetes* ratio in MPA + Probiotic group decreased to 0.45, indicating that probiotics could balance intestinal microflora dysbiosis to attenuate MPA-induced colitis in mice. Meanwhile, according to LefSe analysis, we also found that *Clostridiales*, belonging to *Firmicutes* phylum, was the most significantly different microbiota flora in MPA group compared with CNTL group. In review of literatures, several enteric clostridial diseases also found in humans. Of note, the enteric infections caused by *Clostridium perfringens* and *Clostridium difficile* are the most prevalent ones (McConnie and Kastl, 2017; Uzal et al., 2018). Therefore, *Clostridiales* might be the potential therapy target in MPA-induced colitis and further studies are required to decipher the role of *Clostridiales* in the development of MPA-induced colitis.

Fecal microbiota transplantation (FMT), a treatment aiming to restore dysbiosis by transferring stool from a healthy donor into the patient, has been reported for the treatment of IBD (Fang et al., 2018) or ulcerative colitis (Blanchaert et al., 2019; Costello et al., 2019). FMT could directly change the recipient’s intestinal microbiota to normalize the composition and gain a therapeutic benefit (Wang et al., 2019), but the potential outcome of FMT on MPA-induced colitis have not been explored and elucidated. A randomized clinical trial has indicated that FMT is effective in preventing recurrent *Clostridium difficile* diarrhea (Kao et al., 2017); Considering that *Clostridiales* could be the potential therapy target in MPA-induced colitis, we believe that FMT will have a satisfactory outcome on the treatment of MPA-induced colitis in the future.

## 5. Conclusion

To our knowledge, this is the first study to explore the relationship between probiotics, intestinal microbiota and MPA-induced colitis. Our results showed that severe colitis caused by MPA in mice was linked with intestinal barrier dysfunction and intestinal microbiota dysbiosis. Probiotics could effectively enhance the expression of sIgA, ZO-1 and Occludin in colon and improve intestinal microbiota dysbiosis to ameliorate MPA-induced colitis. *Clostridiales* might be the dominant intestinal microflora and serve as the potential therapy target in MPA-induced colitis. Our findings provide a novel sight into the application of probiotics as an effective agent to ameliorate MPA-induced colitis.

## Data availability statement

The data presented in the study are deposited in the NCBI repository, accession number PRJNA992377.

## Ethics statement

The animal study was reviewed and approved by Committee on the Ethics of Animal Experiments of the Third Xiangya Hospital.

## Author contributions

YN and YM designed the experiment. PZ and JC performed experiments and wrote the first draft of the manuscript. All authors contributed to the article and approved the submitted version.

## Funding

This work was supported by National Natural Science Foundation of China (grant number: 82100695 to PZ) and Natural Science Foundation of Hunan Province (grant number: 2022JJ40759 to PZ).

## Conflict of interest

The authors declare that the research was conducted in the absence of any commercial or financial relationships that could be construed as a potential conflict of interest.

## Publisher’s note

All claims expressed in this article are solely those of the authors and do not necessarily represent those of their affiliated organizations, or those of the publisher, the editors and the reviewers. Any product that may be evaluated in this article, or claim that may be made by its manufacturer, is not guaranteed or endorsed by the publisher.
